# Fish community structure and diversity in the Ningxia section of the main stream of the Yellow River in China

**DOI:** 10.7717/peerj.20228

**Published:** 2025-12-19

**Authors:** Jiacheng Liu, Shuhan Xiong, Peilun Li, Yanbin Liu, Yongjie Wang, Kai Liu, Jilong Wang

**Affiliations:** 1 Heilongjiang River Fisheries Research Institute, Chinese Academy of Fishery Sciences, Harbin, China; 2 Scientific Observing and Experimental Station of Fishery Resources and Environment in Heilongjiang River Basin, Ministry of Agriculture and Rural Affairs, Harbin, China; 3 National Agricultural Experimental Station for Fishery Resources and Environment, Fuyuan, China; 4 Ningxia Fisheries Research Institute, Yinchuan, China

**Keywords:** Yellow River, Fish community structure, Species diversity, Environmental factors, Conservation

## Abstract

The Ningxia section of the Yellow River represents a critical habitat for freshwater fish biodiversity, yet its ecological integrity faces increasing threats from anthropogenic activities. To comprehensively assess the fish community structure, diversity, and its relationship with environmental factors in the Ningxia section of the Yellow River, we conducted seasonal surveys of fish resources and environmental conditions at 15 selected sites from July 2022 to September 2023. Our study employed quantitative analyses to evaluate fish community characteristics, spatiotemporal distribution patterns, and their interactions with environmental variables. A total of 42 fish species, belonging to 34 genera, 11 families, and six orders, were identified, with Cyprinidae being the dominant family (57.14%). Carnivorous species were the most abundant (22 species), followed by omnivorous (16 species) and herbivorous (four species) fish. The Relative Importance Index (*IRI*) identified *Gobio huanghensis* and *Carassius auratus* as the dominant species in this region. Biodiversity indices revealed a mean Margalef richness index of 3.066, Pielou evenness index of 0.5911, Shannon-Wiener diversity index of 1.791, and Simpson dominance index of 0.7083. The Abundance-Biomass Comparison (ABC) curve indicated moderate disturbances to fish communities in spring and autumn, while communities remained stable in summer and winter. Spatially, six sites (Nanchangtan, Shapotou, Jinshawan, Meijiawan, Linhe, and Taole) exhibited significant disturbances, while others showed moderate or stable conditions. The results of cluster analysis and non-metric multidimensional scaling (NMDs) indicated no significant differences in fish community structure among the sampling sections in the Ningxia reach of the Yellow River. Redundancy analysis (RDA) identified water temperature and ammonia nitrogen as the primary environmental factors influencing fish community structure. Our findings highlight the combined impacts of human activities and environmental changes on fish communities in the Ningxia section of the Yellow River. These results provide a scientific basis for the conservation and sustainable management of fishery resources in this ecologically sensitive region.

## Introduction

Fish represent a fundamental component of aquatic biodiversity and play a pivotal role in maintaining ecosystem balance and function in freshwater environments (Park et al., 2006). As key indicators of ecosystem health, fish community diversity indices provide valuable insights into both structural characteristics of riverine assemblages and their responses to ecological gradients (Buisson et al., 2008). A comprehensive understanding of fish community dynamics encompassing spatial and temporal patterns of diversity, stability, and environmental sensitivity is critical for advancing effective fisheries management and aquatic conservation efforts (Pease et al., 2011; He et al., 2016).

The Ningxia section of the Yellow River mainstem is located in the river’s upper reaches, extending from Nanchangtan Village in Zhongwei City to Huinong District in Shizuishan City. Spanning approximately 397 km, this segment has an altitude range of 1,090–1,300 m (Qi, 2017). Geomorphologically, Xiaheyan Village (Challe Town, Zhongwei City) serves as a natural boundary dividing this segment into two distinct reaches: (1) an upstream canyon section characterized by narrow valleys and steep gradients, and (2) a downstream alluvial plain featuring wider channels and extensive floodplains (He et al., 2022). Compared to the upper reaches, the Ningxia section features a relatively moderate flow velocity, richer availability of prey organisms, and higher fish diversity (Hu et al., 2022). This fluvial ecosystem supports critical spawning and nursery habitats for multiple endemic fish species, including *Coreius septentrionalis*, *Chuanchia labiosa*, *Rhinogobio nasutus*, and *Triplophysa siluroides* (Zhang, Shen & Li, 2011).

In recent years, fish biodiversity in the study area has significantly declined due to anthropogenic disturbances, including hydropower project construction and deterioration of water environments. Many indigenous fish populations have been severely impacted, with the *C. septentrionalis* having not been recorded in this river section for nearly 30 years (Liu et al., 2010). Additionally, the invasion of non-native fish species has further disrupted the local fish community. Therefore, the conservation and restoration of fish populations have become a top priority. Currently, research on the fish community structure and diversity in this water area remains scarce. Therefore, this study conducted a comprehensive seasonal survey of fish populations across 15 sampling stations in the Ningxia section. The objectives were to (1) document fish species composition and spatiotemporal distribution patterns, (2) assess changes in fish community resources, and (3) evaluate the relationship between fish population structure and environmental variables. This study aims to provide fundamental data for the management and conservation of fish resources in this critical aquatic ecosystem.

## Materials and Methods

### Access to data

**Figure 1:**
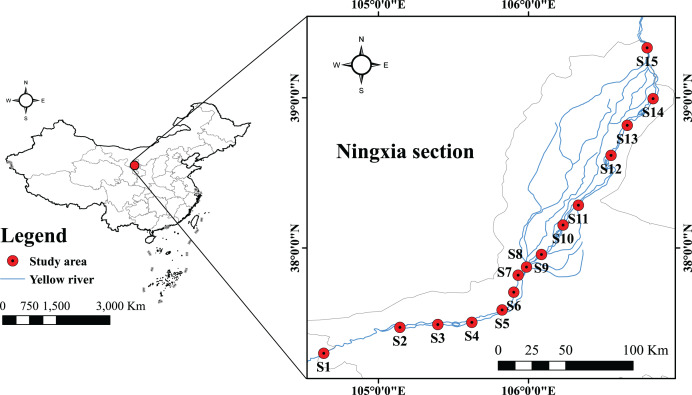
Location map of sampling points for the Yellow River in Ningxia.

Fisheries resources and environmental surveys were conducted in the Ningxia section of the Yellow River mainstream in July 2022, as well as in February, May, and September 2023. Survey methodologies were performed in accordance with the Manual of Investigation for Fisheries Resources in Inland Waters (Zhang & He, 1991). Following field investigations and integration of site visit findings, 15 sampling sections were finally chosen for examination (Table 1) (Fig. 1). Three types of fishing nets were deployed: ground cages (mesh size 0.5 cm, dimensions 20 m × 0.4 m × 0.4 m), fixed composite gillnets (mesh sizes 4, 6, 8 cm, with a height of 2 m and length of 20 m), and drift gillnets (mesh sizes 2, 4, 6 cm, with a height of 2 m and length of 30 m). The ground cages and fixed gillnets were typically set for 24 h, while the drift gillnets were deployed over a drifting distance of 2 km. Refer to relevant materials (Wu & Wang, 2008; Cai, Zhang & Wang, 2013; Li, 2017) to identify the species of fish collected. Fish samples unable to be identified *in situ* were fixed in 5–10% formalin solution, with capture time and location recorded, and subsequently transported to the laboratory for species identification. For biological measurements of the catches, body length was measured to the nearest 1 mm, and weight to the nearest 0.01 g. During the survey, water temperature, dissolved oxygen (DO), pH, chlorophyll a (Chl-a), and ammonia nitrogen (NH_3_-N) were measured using a YSI portable multi-parameter water quality analyzer. Additionally, 1 L water samples were collected from each section, cryopreserved, and subsequently analyzed for NH_3_-N and Chl-a concentrations. All sampling procedures strictly adhered to the guidelines provided by Heilongjiang River Fisheries Research Institute of Chinese Academy of Fishery Sciences for Laboratory Animal Welfare and Ethical Review (No: 20220420-004).

**Table 1:** Coordinates of sampling station in Ningxia section of Yellow River.

Sampling station	number	Longitude	Latitude
Nanchangtan	S1	E 104.389	N 36.552
Shapotou	S2	E 104.676	N 36.681
Yuding	S3	E 105.307	N 37.472
Baima	S4	E 105.545	N 37.482
Mingsha	S5	E 105.816	N 37.563
Niushoushan	S6	E 105.988	N 37.839
Huangshawan	S7	E 106.152	N 37.972
Kushou	S8	E 106.264	N 38.282
Meijiawan	S9	E 106.357	N 38.556
Wanghong	S10	E 106.448	N 38.679
Linhe	S11	E 106.505	N 38.775
Yueyahu	S12	E 106.663	N 38.836
Taole	S13	E 106.831	N 38.952
Hongyazi	S14	E 106.754	N 39.302
Wayaocun	S15	E 106.773	N 39.352

### Data processing and analysis

#### Biodiversity

The Shannon-Wiener diversity index (*H*), Margalef species richness index (*D*), Pielou evenness index (*E*) and Simpson dominance index (*C*) (Gao et al., 2017a) were employed to assess the species diversity of fishes in the Ningxia section of the Yellow River mainstream.

(1) $$H = - \mathop \sum \limits_{{\rm i} = 1}^{\rm S} ({{\rm n}_{\rm i}}/{\rm N}){\rm ln}\left( {{{\rm n}_{\rm i}}/{\rm N}} \right)$$

(2) $$D = \displaystyle{{S - 1} \over {\ln N}}$$

(3) $$E = \displaystyle{H \over {{\rm lnS}}}$$

(4) $$C = 1 - \mathop \sum \limits_{i = 1}^s {\left( {\displaystyle{{{n_i}} \over N}} \right)^2}$$

where *n*_*i*_ represents the quantity of the *i* species, *N* is the number of all species in the community, and *S* is the number of species in the community.

#### Community-dominant species

Index of relative importance (*IRI*) was used to calculate the dominant species in fish communities.

(5) $$IRI = \left( {N{\rm \% } + W{\rm \% }} \right) \times F\rm \%$$

where *N*% is the percentage of the number of fish in the total number of fish, *W*% is the percentage of the biomass of a certain fish in the total biomass, and *F*% is the percentage of the frequency of a certain fish in the total frequency. Define the species with an *IRI* value greater than 1,000 as the dominant species (Li et al., 2013b).

#### Ecological type division

Following relevant literature (Li, 2017), the ecological types of collected fishes were classified. Based on habitat preferences, they were categorized into three types: demersal sedentary species, pelagic species, and semi-pelagic species. According to feeding habits, the fishes were further divided into three groups: omnivorous, carnivorous, and herbivorous.

#### Community stability

The Abundance-Biomass Comparison (ABC) curves and W statistics were employed to characterize community stability and evaluate the dominance of biomass over abundance (*via* the *W*-statistic). In aquatic communities, ABC curves serve as robust indicators of habitat quality. The W statistic, ranging between −1 and 1, provides critical insights into community structure: When *W* > 0, the biomass curve lies above the abundance curve, signifying that the community is dominated by one or more large fish species. This pattern typically reflects minimal environmental disturbance. When *W* < 0, the biomass curve falls below the abundance curve, indicating dominance by small fish species and suggesting significant disturbance impacts. An intersection of the biomass and abundance curves denotes a moderate disturbance regime within the community (Stojković Piperac, Milošević & Simić, 2015). The ABC curves were generated using Primer 6.0 software.

(6) $$W = \sum \left( {{B_i} - {A_i}} \right)/50\left( {S - 1} \right)$$

where *B*_*i*_ and *A*_*i*_ are the cumulative percentages of biomass and quantity corresponding to species serial numbers in the *ABC* curve, respectively, and *S* is the number of species present.

#### Community structure similarity analysis

Using Primer 6.0 software, we constructed a similarity matrix based on the relative abundance of fish species across sampling stations, with species designated as the abscissa and stations as the ordinate. The Bray-Curtis similarity coefficient was applied to quantify dissimilarity among communities. To visualize spatial patterns in fish assemblage structure, we performed hierarchical cluster analysis and non-metric multidimensional scaling (NMDS). The reliability of the NMDS ordination was assessed using the stress coefficient, with lower values indicating better ordination fit (Fang et al., 2023). Following conventional interpretation guidelines, stress values <0.2 provide meaningful ecological interpretation, values <0.1 indicate good ordination quality, and values <0.05 demonstrate highly representative results (Yu et al., 2021).

#### Relationship between fish community structure and water environmental factors

To minimize the impact of rare and incidental species on research outcomes, this study only used dominant fish species to investigate the relationships between fish community structure and water environmental variables in the Ningxia section of the Yellow River. Detrended correspondence analysis (DCA) was performed using Canoco 5.0 software (Lepš & Šmilauer, 2014). The selection of the appropriate analysis method was guided by the gradient length (LGA) of each axis. Redundancy analysis (RDA) is preferred when the LGA value is less than 3, whereas canonical correspondence analysis (CCA) is applied when the LGA exceeds 4. Based on the outcomes of the DCA, RDA was performed in this study. Furthermore, a Monte Carlo test was employed to evaluate the significant effects of environmental factors on variations in the fish community.

## Results

### Fish species composition, ecological type and dominant species

The study results showed that a total of 42 fish species were collected from the Ningxia section of the Yellow River mainstem, belonging to six orders, 11 families, and 34 genera. The Cyprinidae family was the most species-rich, with 24 species accounting for 57.14% of the total. Among the 42 species recorded, 25 were non-native species (59.5%), while the remaining 17 were native species (40.5%). According to feeding habits, the fish were classified into different ecological types: carnivorous species dominated with 22 species (52.4%), followed by omnivorous species with 16 species (38.1%), and herbivorous species with four species (9.5%). In terms of habitat, benthic fish were the most prevalent, comprising 25 species (59.5%), followed by pelagic fish with nine species (21%) and semi-pelagic fish with eight species (19%) (Table 2).

**Table 2:** Species composition and ecological classification of fish collected in each section of the Ningxia section of the main stream of the Yellow River.

Species	S1	S2	S3	S4	S5	S6	S7	S8	S9	S10	S11	S12	S13	S14	S15	Ecological types		Exotic species
																Feeding habits	Habit characteristics	
*Cypriniformes*																		
*Cyprinidae*																		
*Gobio huanghensis*	+	+	+	+	+	+	+	+	+	+	+	+	+	+	+	C	D	
*Abbottina rivularis*			+	+	+	+	+	+	+	+	+	+	+	+	+	O	D	*
*Pseudorasbora parva*	+	+	+	+	+	+	+	+	+	+	+	+	+	+	+	O	L	
*Gobio rivuloides*											+					C	D	
*Romanogobio tenuicorpus*															+	C	D	
*Rhinogobio nasutus*											+	+		+	+	C	D	
*Hemibarbus maculatus*										+		+	+	+	+	C	L	*
*Ctenopharyngodon idellus*					+		+	+	+	+	+		+	+	+	H	L	*
*Aristichthys nobilis*								+					+			O	U	*
*Squaliobarbus curriculus*		+							+	+		+	+	+		O	L	
*Leuciscus chuanchicus*		+	+	+	+	+	+	+	+	+	+	+	+		+	O	U	
*Rhodeus ocellatus*			+	+	+	+	+	+		+	+	+	+	+	+	H	L	*
*Rhodeus sinensis*		+	+	+	+	+	+	+		+	+	+	+	+	+	H	L	*
*Acanthorhodeuschankaensis*	+		+	+	+		+	+	+	+	+	+	+	+	+	H	L	*
*Cyprinus carpio*	+	+	+	+	+	+	+	+	+	+	+	+	+		+	O	D	
*Cyprinus carpiovar*											+		+			O	D	*
*Carassius auratus*	+	+	+	+	+	+	+	+	+	+	+	+	+	+	+	O	D	
*Hypophthalmichthys molitrix*						+	+	+			+	+	+		+	O	U	*
*Aphyocypris chinensis*													+		+	O	U	
*Cultrichthys erythropterus*											+	+	+	+	+	C	U	*
*Culter alburnus*														+	+	C	U	*
*Hemiculter* *leucisculus*				+	+	+	+		+	+	+	+	+	+	+	O	U	*
*Magalobrame terminalis*													+			O	L	*
*Gobiobotia pappenheimi*									+			+		+		C	D	
*Cobitidae*																		
*Triplophysa pappenheimi*		+	+						+	+						C	D	
*Lefua costata*	+		+					+							+	O	D	*
*Misgurnus* *anguillicaudatus*			+	+				+		+		+	+			O	D	
*Cobitis granoei*			+		+								+			O	D	
*Paramisgurnus* *dabryanus*		+	+		+		+	+		+		+	+		+	O	D	*
*Triplophysa* *siluroides*	+															C	D	
*Perciformes*																		
*Gobiidae*																		
*Rhinobius giurinus*			+	+	+	+	+	+	+	+	+	+	+	+		C	D	*
*Rhinobius cliffordpopei*			+	+	+		+			+	+	+	+			C	D	*
*Eleotridae*																		
*Hypseleotris swinhonis*	+		+	+	+	+	+	+		+	+		+	+	+	C	D	*
*Percichthyidae*																		
*Sander lucioperca*													+			C	D	*
*Perca fluviatilis*			+	+	+	+	+	+					+			C	D	*
*Channidae*																		
*Channa argus*					+	+		+					+			C	D	*
*Salmoniformes*																		
*Salmonidate*																		
*Salmo irideus*	+															C	D	*
*Osmeridae*																		
*Hypomesus* *olidus*	+	+	+	+	+	+	+	+	+	+		+	+	+	+	C	U	*
*Siluriformes*																		
*Siluridae*																		
*Silurus asotus*	+	+					+	+	+		+	+	+	+	+	C	D	
*Silurus lanzhouensis*	+	+	+	+	+	+	+	+	+	+	+	+	+	+	+	C	D	
*Acipenseriformes*																		
*Acipenseridae*																		
*Acipenser*										+	+	+				C	D	*
*Crprinodntifprmes*																		
*Adrianichthyidae*																		
*Oryzias latipes*				+	+	+							+			C	U	*

**Note:**

(1) S1 is Nanchangtan, S2 is Shapotou, S3 is Yuding, S4 is Baima, S5 is Mingsha, S6 is Niushoushan, S7 is Jinshawan, S8 is the Kushou, S9 is Meijiawan, S10 is Wanghong, S11 is Linhe, S12 is Yueyahu, S13 is Taole, S14 is Hongyazi, S15 is Wayaocun. (2)* Exotic species (3) Ecological type: U, Upper; L, Lower; D, Demersal; C, Carnivorous; O, Omnivorous; H, Herbivore.

In the Ningxia section of the Yellow River, *Gobiobotia huanghensis* emerged as the overall dominant species. Temporal analysis revealed distinct seasonal and interannual variations in dominant species composition across the four sampling campaigns, with *Carassius auratus*, *Cyprinus carpio*, and *G. huanghensis* maintaining consistent dominance throughout all surveys. The July 2022 survey recorded additional dominance by *Pseudorasbora parva* and *Squalidus lanzhouensis*, while February 2023 was characterized by *Leuciscus chuanchicus* and *Abbottina schankaensis*. By May 2023, *A. schankaensis* and *P. parva* (the latter continuing its dominance from previous months) predominated, followed by *L. chuanchicus*, *A. schankaensis*, and *Rhinogobius giurinus* in September 2023. Spatial analysis demonstrated significant geographic heterogeneity in dominant species distribution across sampling stations, with *G. huanghensis* and *C. carpio* showing widespread dominance. Notably, *G. huanghensis* maintained dominance across all 15 surveyed sections, demonstrating remarkable spatial consistency (Table 3).

**Table 3:** Dominant fish species in each sampling section of the Ningxia section of the Yellow River.

Sites	Dominant species
S1	*G. Huanghensis*, *C. carpio*, *S. asotus*.
S2	*G. Huanghensis*, *C. carpio*.
S3	*G. Huanghensis*, *C. carpio*, *Pseudorasbora parva*, *Carassius auratus*.
S4	*G. huanghensis*, *C. carpio*, *P. parva*, *L. chuanchicus*, *S. lanzhouensis*.
S5	*G. Huanghensis*, *C. carpio*, *L. chuanchicus*, *P. parva*.
S6	*G. Huanghensis*, *C. carpio*, *L. chuanchicus*, *P. parva*.
S7	*G. Huanghensis*, *C. carpio*, *L. chuanchicus*, *P. parva*, *H. molitrix*.
S8	*G. huanghensis*, *C. carpio*, *P. parva*.
S9	*G. Huanghensis*, *C. carpio*.
S10	*G. Huanghensis*, *L. chuanchicus*, *S. lanzhouensis*, *A. deuschankaensis*, *H. leucisculus*.
S11	*A. Deuschankaensis*, *R. ocellatus*, *C. auratus*, *S. lanzhouensis*, *G. huanghensis*.
S12	*G. Huanghensis*, *L. chuanchicus*, *C. carpio*, *C. auratus*.
S13	*L. Chuanchicus*, *C. carpio*, *C. auratus*, *P. parva*.
S14	*G. Huanghensis*, *C. auratus*, *P. parva*.
S15	*G. Huanghensis*, *S. lanzhouensis*, *C. carpio*, *C. auratus*.

### Fish diversity

According to the survey results, the Shannon-Wiener diversity index (*H*) exhibited values ranging from 1.189 to 2.441 across sampling sites, with an average of 1.791. The lowest index was recorded at Station S1, while Station S14 showed the highest. For the Margalef species richness index (*D*), values varied between 2.112 and 3.805 (mean = 3.066), with the lowest richness observed at Station S2 and the highest at Station S12. The Pielou evenness index (*E*) spanned 0.4505–0.7897 (mean = 0.5911), with the minimum and maximum values recorded at Station S1 and Station S14, respectively. The Simpson dominance index (*C*) ranged from 0.4536 to 0.8802 (mean = 0.7083), with the smallest value at Station S1 and the largest at Station S14.

From a temporal perspective, the Shannon-Wiener diversity index (*H*) fluctuated between 2.012 and 3.132 across the four surveys, with an average of 2.474. The Margalef species richness index (*D*) ranged from 2.213 to 3.588 (mean = 2.89), while the Pielou evenness index (*E*) varied between 0.5214 and 0.7356 (mean = 0.6456). The Simpson dominance index (*C*) showed the widest range, spanning 0.62 to 0.9644 with an average of 0.7177 (Table 4).

**Table 4:** Fish diversity indices in sampling sections of the Ningxia section of the Yellow River.

Sites	Shannon-Wiener (*H*)	Margalef (*D*)	Pielou (*E*)	Simpson (*C*)
S1	1.189	2.666	0.4505	0.4536
S2	1.44	2.112	0.5614	0.5906
S3	2.169	2.853	0.7366	0.8438
S4	1.543	2.506	0.5240	0.62
S5	1.644	3.389	0.5318	0.6417
S6	1.490	2.142	0.5502	0.6678
S7	1.76	2.971	0.5693	0.7429
S8	1.738	3.078	0.5542	0.7195
S9	1.386	2.977	0.4795	0.5188
S10	2.258	3.586	0.7201	0.8291
S11	1.847	3.034	0.5975	0.7768
S12	2.104	3.805	0.6536	0.7898
S13	1.916	3.555	0.5579	0.7697
S14	2.441	3.564	0.7897	0.8802
S15	1.947	3.752	0.5907	0.7808

### Abundance biomass comparison curve

**Figure 2:**
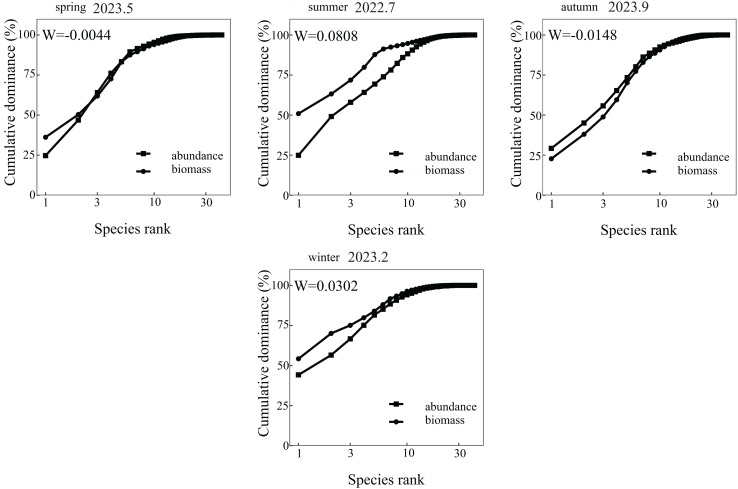
Analysis of Abundance/Biomass curves in each sampling season of the Ningxia section of the Yellow River Mainstem.

**Figure 3:**
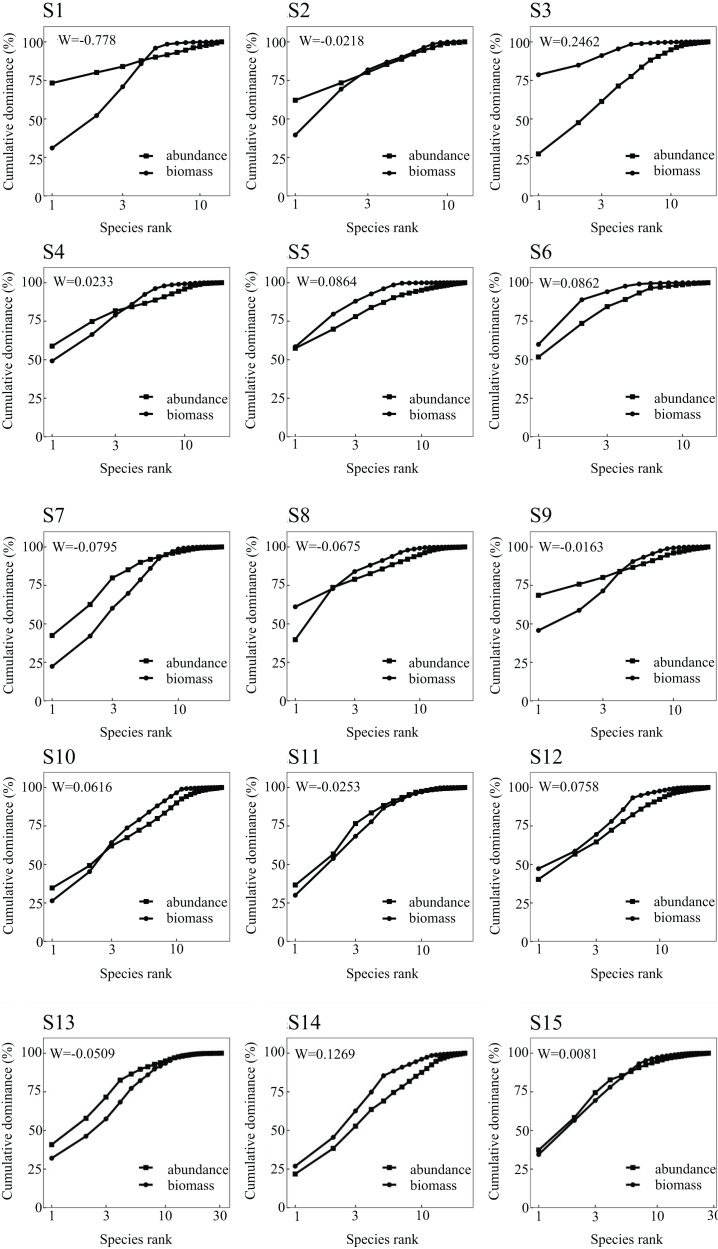
Abundance/biomass curve analysis of each sampling section of the Ningxia section of the main stream of the Yellow River.

The ABC curve indicates that, over the course of 2023, the *W* values in May and September were less than zero. During these months, the abundance and biomass curves intersected, suggesting that the fish community experienced moderate disturbances. Conversely, the *W* values in July 2022 and February 2023 exceeded zero, with the biomass curve positioned above the abundance curve, pointing to a stable state in the fish community during these periods (Fig. 2). From a spatial perspective, the *W* values at Nanchangtan (S1), Shapotou (S2), Jinshawan (S7), Meijiawan (S9), Linhe (S11), and Taole (S13) were all below zero. Typically, the abundance dominance curve was above the biomass dominance curve, with the starting point of the former higher than that of the latter, indicating significant disturbances within the fish communities at these six stations. Conversely, the *W* values at Baima (S4), Kushou (S8), Wanghong (S10), and Wayaocun (S15) were greater than zero, where the biomass dominance curve intersected with the abundance dominance curve, signaling moderate disturbances at these four stations. Meanwhile, the *W* values at Yuding (S3), Mingsha (S5), Niushoushan (S6), Yuyahu (S12), and Hongyazi (S14) also exceeded zero, with the biomass dominance curve consistently above the abundance dominance curve, and the starting point of the biomass dominance curve surpassed that of the abundance dominance curve, reflecting a relatively stable fish community structure at these five sites (Fig. 3).

### Community cluster analysis

**Figure 4:**
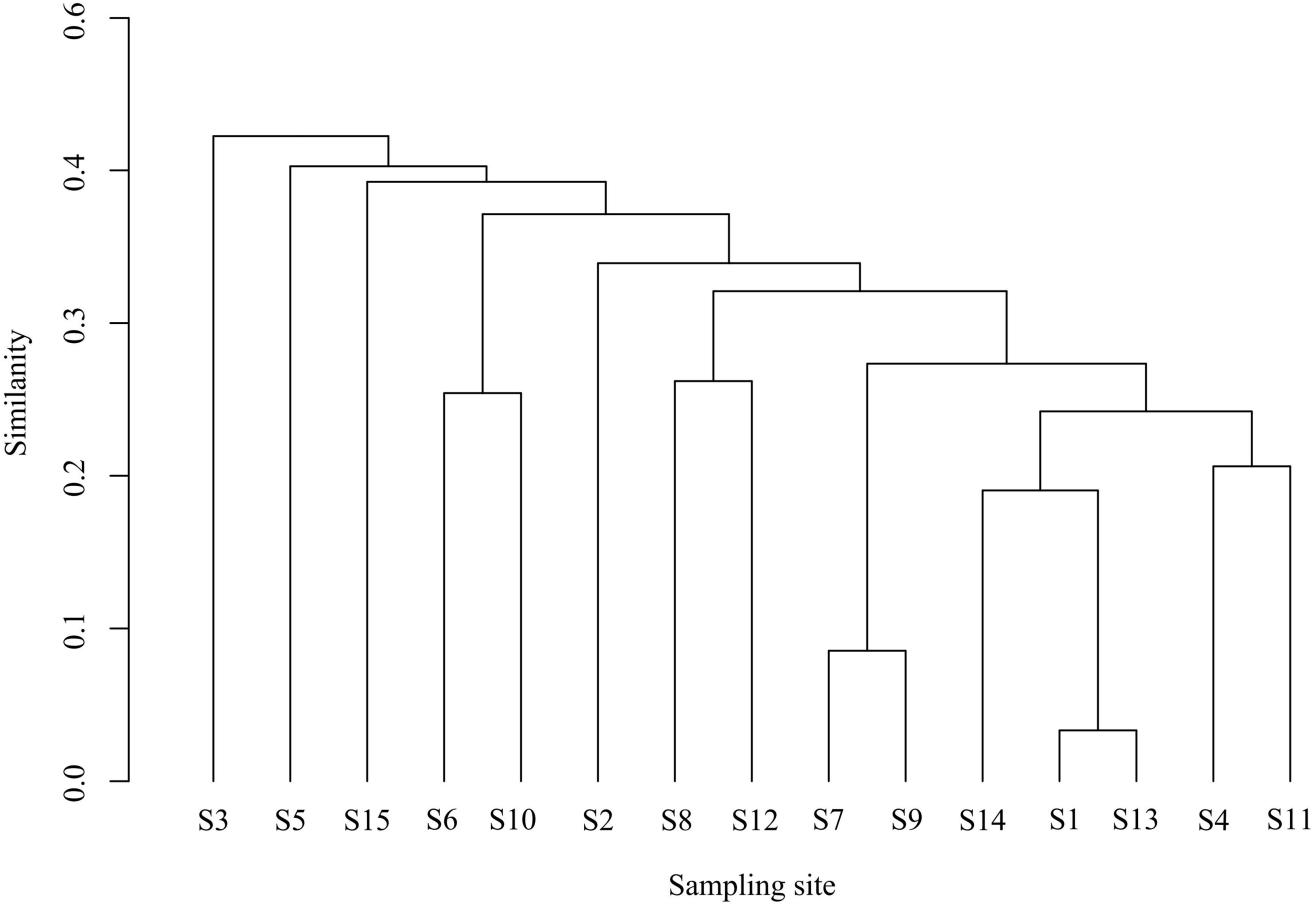
Bray-cluster analysis map of each sampling section of the Ningxia section of the main stream of the Yellow River.

Cluster and NMDS analyses were conducted on fish communities, as depicted in Fig. 4. Cluster analysis revealed that fish community structures in the Ningxia section of the Yellow River mainstem were grouped into four clusters at a Bray-Curtis similarity level of 48.62%. Notably, visual inspection of cluster dendrograms suggested limited differentiation among sampling sections. One-way analysis of similarities (ANOSIM) tests further indicated no significant differences in community structure among the four clusters (*R* = 1, *P* = 0.1), supporting the hypothesis of homogeneous community composition. The NMDS ordination (stress = 0.07) aligned with cluster results, demonstrating moderate explanatory power for inter-sample relationships.

### Relationship between fish community and water environmental factors

**Figure 5:**
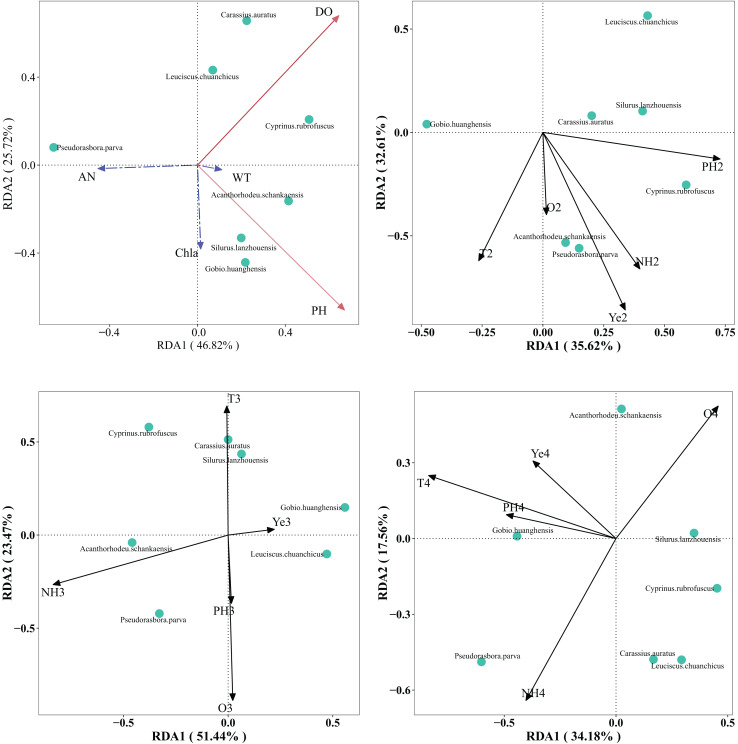
RDA analysis ranking diagram of fish community structure and water environment factors in the Ningxia section of the main stream of the Yellow River.

Note: a is July 2022, b is February 2023, c is May 2023, d is September 2023. WT is water temperature, DO is dissolved oxygen, AN is ammonia nitrogen, Chla is chlorophyll a. The red solid line indicates a significant impact, while the blue dashed line indicates no significant impact.

RDA was performed to explore the relationships between seven dominant fish species (*e.g*., *G. huanghensis*, *C. carpio*) and five water environmental variables (water temperature, DO, pH, Chl-a, and NH_3_-N monitored across the Ningxia section of the Yellow River mainstem. Random permutation tests uncovered significant temporal variations in how water environmental factors influenced fish community structure in the Ningxia section of the Yellow River mainstem. Specifically, water temperature and dissolved oxygen were significant factors in July 2022 (*P* < 0.05); NH_3_-N and Chl-a in February 2023 (*P* < 0.05); NH_3_-N and dissolved oxygen in May 2023 (*P* < 0.05); and only water temperature in September 2023 (*P* < 0.05). It is evident that water temperature and NH_3_-N are the primary environmental factors affecting the fish community in this section of the Yellow River (Fig. 5).

## Discussion

### Status of fish resources

The fish collected in this survey were predominantly cyprinid species, a prevalence that aligns with the common characteristic of freshwater fish faunas across China (Chen, Chen & He, 2002). Compared with historical records of 18 species (He, Wang & Song, 1963) and 26 species (Hu et al., 2022) in the Ningxia section of the Yellow River, the fish species composition identified in this study exhibited higher diversity. This higher diversity may be attributed to more comprehensive survey coverage and more diverse fish sampling methods employed in this study. The shifts in fish ecological types are closely linked to river flow velocity, nutrient levels, and the distribution of aquatic organisms (Li et al., 2013a). In this survey, carnivorous and omnivorous fish dominated the assemblage, comprising 90.5% of the total catch—a notable increase compared to historical records. It is speculated that the reasons for these changes may be water nutrient input from irrigation return flows in the Ningxia section of the Yellow River, loss of fish habitats due to changes in hydrological conditions caused by the construction and operation of the Qingtongxia Reservoir, and continuous decline in water quality, food resources, and habitat conditions caused by high sediment content in the Ningxia section of the Yellow River (Qi, 2017). These factors collectively result in a sustained decline in sensitive fish populations and a corresponding increase in tolerant species. Carnivorous and omnivorous fish, characterized by broader ecological niches and enhanced adaptability to disturbed environments, exhibit greater resilience under these altered conditions (Meng et al., 2022).

Studies indicate that exotic fish generally exhibit higher adaptability than indigenous species, conferring them significant advantages in food acquisition, habitat utilization, and survival-reproduction strategies (Ba & Chen, 2012). Chen, Yang & Li (1998) documented that invasive exotic fish species represent a key driver of endangerment and even extinction of indigenous fish populations on the Yunnan-Guizhou Plateau. In this survey, 25 exotic fish species were recorded, comprising a proportion exceeding that of indigenous species. Among them, top predators like *S. lucioperca* and *C. argus* which occupy high trophic positions in the food web (Li, 2017) exert significant predation pressure on indigenous fish, potentially threatening their populations and biomass. Due to religious practices and artificial stocking, certain non-native species such as *H. olidus* and *Protosalanx hyalocranius* has established naturalized populations in the mainstem of the Yellow River, China (Wang, 2021). Therefore, greater attention should be paid to the potential risks posed by non-native fish species, and rigorous risk assessments should be proactively implemented.

Fish miniaturization has emerged as a widespread phenomenon in inland rivers, reservoirs, and lakes across China (Liu et al., 2021). The results of *IRI* analysis indicated that the dominant fish species in the Ningxia section of the Yellow River mainstem were small-bodied fishes, including *G. huanghensis*, *C. auratus*, and *P. parva*, the predominance of *Cyprinus carpio* in the Ningxia section of the Yellow River primarily results from extensive stocking programs conducted by local fishery management agencies. Potential drivers of this pattern include overfishing, aquatic ecological degradation, and invasion of alien species (Xu et al., 2012; Chen et al., 2012).

### Characteristics of fish communities

Xu et al. (2007) demonstrated a significant positive correlation between water temperature and fish diversity indices. In this study, fish diversity in the Ningxia Yellow River mainstem exhibited marked spatial and seasonal variations. Specifically, biomass, species richness, Shannon-Wiener diversity index, and Margalef richness index in July 2022 were significantly higher than those in the other three surveys, which may be linked to the higher water temperatures in July. On a spatial scale, fish community metrics in the Ningxia Yellow River mainstem showed that the Shannon-Wiener diversity index, Pielou evenness index, and Simpson dominance index were lowest at station S1 and highest at station S14, which may be due to the gentle water flow, suitable habitats, and well-developed coastal shallow waters at station S14 providing optimal spawning and foraging grounds for fish, while station S1 is located in a canyon area with turbulent currents and low water transparency that create harsh conditions for diverse fish assemblages; higher fish species diversity, richness, and evenness indices collectively reflect greater complexity and stability of fish community structures in aquatic environments, where elevated richness and evenness indicate a more balanced species distribution and enhanced community resilience to environmental perturbations (Cao, Wang & Zhang, 2003). Overall, the relatively high average richness index (3.066) and evenness index (0.59) of fish in the Ningxia Yellow River mainstem suggest a complex and stable fish community structure in this section.

The ABC curve can effectively determine the degree of disturbance to fish communities (Yemane, Field & Leslie, 2005). In this study, the fish communities surveyed in July 2022 and February 2023 showed no signs of disturbance, with the *W* value reaching its highest level among the four surveys in July 2022. This may be attributed to the recent conclusion of the fishing ban period in the Ningxia Yellow River section, as the months-long prohibition provided effective protection for local fishery resources. In contrast, February 2023 coincided with winter, when low water temperatures led to reduced fish activity, consequently resulting in undisturbed community structure during this sampling period. From a spatial perspective, cross-sections with varying degrees of disturbance are dominated by small fish species, whose ecological habits are generally adaptable and pollution-tolerant. Large fish species—primarily *H. molitrix*, *A. nobilis*, *C. idellus* and *C. carpio* are scarce and predominantly consist of cultured and released individuals, coinciding with river sections experiencing varying levels of water pollution. In Yuding (S3), Mingsha (S5), Niushoushan (S6), Yueyahu (S12), and Hongyazi (S14) where fish community structures were relatively stable the water quality was comparatively optimal, and large-bodied fish comprised a high proportion of the catch.

The analysis results of Cluster indicate that, there is no significant difference in the fish community structure of most sections of the Ningxia section of the Yellow River main stream. The structure of fish communities is closely related to the water environment they inhabit (Fang et al., 2023). The water environments they inhabit are similar, leading to a high degree of similarity in fish community composition (Li et al., 2012). Most sections of the Ningxia Yellow River feature gentle water flow, and their substrata include both sediments and gravel beaches, resulting in relatively high habitat heterogeneity (Qi, 2017).

### Relationship between fish communities and water environmental factors

RDA analysis can effectively elucidate the relationships between fish communities and water environmental factors, revealing the correlations between fish species and distinct habitat types (Zhang, 2011). The distribution and characteristics of fish community structure are influenced not only by the intrinsic ecological habits of fish but also by water environmental factors that shape their survival conditions (Liu et al., 2021). Water environmental factors influencing fish community structural characteristics are complex and diverse, including water temperature, DO, pH, and NH_3_-N, among others (Wang et al., 2013; Wang, 2013; Wu et al., 2014). The results of RDA indicated that water temperature was the primary water environmental factor influencing the fish community structure in the Ningxia section of the Yellow River mainstem during July 2022 and September 2023. As a critical driver of fish community diversity, water temperature significantly affects fish growth, development, population reproduction, and other life-history traits. With increasing water temperature, fish exhibit enhanced metabolism of prey resources, accelerated growth and development rates, and subsequent increases in population size (Jacob et al., 1998). And this result is consistent with the research conducted by Gao et al. (2017b) on the fish community in Beijiang and Li (Li, Li & Jia, 2010) on the fish community in Xijiang, both of which reflect the significant influence of water temperature on the structure of fish communities.

Excessive NH_3_-N content in water bodies is an important factor leading to eutrophication (Qian et al., 2021). In water bodies with high eutrophication levels, the proportion of large-bodied fish gradually decreases, while the proportion of small-bodied fish continues to increase (Tammi et al., 1999). The results of this study show that small-bodied fish are the dominant species in the fish community of the Ningxia section of the Yellow River, which may be partially attributed to the excessive ammonia nitrogen content in the water (Qi, 2017). The results of RDA analysisfurther revealed that ammonia nitrogen was the primary water environmental factor influencing the fish community structure in the Ningxia Yellow River mainstem during February and May 2023. This finding is consistent with the research by Fang et al. (2023). On fish communities in the middle reaches of the Yangtze River, which reflects the significant influence of ammonia nitrogen concentration in water on fish community structure.

## Conclusion and suggestions

This study provides the first comprehensive assessment of fish community structure and dynamics in the Ningxia section of the Yellow River, revealing 42 species dominated by carnivorous-omnivorous cyprinids (90.5%) with significant non-native representation (59.5%). Key findings demonstrate: (1) Persistent dominance of *G. huanghensis* alongside spatiotemporal shifts in co-dominant species; (2) Moderate ecological disturbance at 40% of sampling stations *via* ABC curve analysis; (3) Water temperature and ammonia nitrogen as primary environmental drivers of community structure *via* RDA; and (4) Pronounced miniaturization trends linked to stocking practices (*Cyprinus carpio*) and eutrophication. These results establish critical baselines for conservation in this ecologically vulnerable reach, highlighting the urgent need to mitigate anthropogenic impacts—particularly invasive species proliferation and nutrient loading. Future research should prioritize: (i) Quantifying trophic cascades from invasive predators (*e.g*., *S. lucioperca*), (ii) Assessing dam operations (Qingtongxia Reservoir) on habitat fragmentation, and (iii) Developing size-based ecosystem models to forecast community trajectories under climate change scenarios.

## Supplemental Information

10.7717/peerj.20228/supp-1 Supplemental Information 1 Data on catch dominant species and diversity indices for each section and season.

10.7717/peerj.20228/supp-2 Supplemental Information 2 Data of NMDS.

10.7717/peerj.20228/supp-3 Supplemental Information 3 Data of RDA.

## Additional Information and Declarations

### Data Availability

The following information was supplied regarding data availability:

The raw data is available in the Supplemental Files.
